# QUANTITATIVE RELAXOMETRY APPROACHES TO CHARACTERIZING BRAIN AGING: APPLICATIONS ACROSS THE LIFESPAN

**DOI:** 10.1093/geroni/igad104.1281

**Published:** 2023-12-21

**Authors:** Doug Dean

**Affiliations:** University of Wisconsin, Madison, Wisconsin, United States

## Abstract

Quantitative magnetic resonance imaging (qMRI) aims to measure meaningful physical or chemical parameters that are sensitive to and that can be used to characterize the underlying tissue microstructure. The quantitative nature of these data suggest that derived measures are ideally reproducible within and across different sites, allowing qMRI data to be more readily compared across different anatomical regions, among different subjects, and across time. The longitudinal (T1) and transverse (T2) relaxation times are fundamental to the measured MRI signal and, in the brain, highly sensitive to several anatomical changes, including changes of free water, iron content, and brain myelination. Thus, measurement of these relaxation times can be used to characterize the underlying tissue microstructure and can provide insight into the underlying processes of brain development and aging. As such, there is growing interest in using relaxometry-based techniques for a broad range of clinical and research applications. In this presentation, I will discuss recent advancements in quantitative relaxometry approaches and describe how these techniques are being used to gain a better understanding of the highly dynamic and nonlinear mechanisms that support brain development and aging.

